# SRRF: Universal live-cell super-resolution microscopy

**DOI:** 10.1016/j.biocel.2018.05.014

**Published:** 2018-08

**Authors:** Siân Culley, Kalina L. Tosheva, Pedro Matos Pereira, Ricardo Henriques

**Affiliations:** a MRC Laboratory for Molecular Cell Biology, University College London, Gower Street, London WC1E 6BT, UK; b Department of Cell and Developmental Biology, University College London, Gower Street, London, WC1E 6BT, UK; c The Francis Crick Institute, 1 Midland Road, London, NW1 1AT, UK

**Keywords:** Super-resolution microscopy, Fluorescence, Image processing, Live-cell imaging

## Abstract

Super-resolution microscopy techniques break the diffraction limit of conventional optical microscopy to achieve resolutions approaching tens of nanometres. The major advantage of such techniques is that they provide resolutions close to those obtainable with electron microscopy while maintaining the benefits of light microscopy such as a wide palette of high specificity molecular labels, straightforward sample preparation and live-cell compatibility. Despite this, the application of super-resolution microscopy to dynamic, living samples has thus far been limited and often requires specialised, complex hardware. Here we demonstrate how a novel analytical approach, Super-Resolution Radial Fluctuations (SRRF), is able to make live-cell super-resolution microscopy accessible to a wider range of researchers. We show its applicability to live samples expressing GFP using commercial confocal as well as laser- and LED-based widefield microscopes, with the latter achieving long-term timelapse imaging with minimal photobleaching.

## Highlights

• SRRF is a purely analytical super-resolution microscopy approach available as an open-source easy-to-use plugin for ImageJ.
• SRRF is compatible with any fluorophore, including conventional fluorescent proteins such as GFP.
• SRRF can be used to retrieve super-resolution information from most common fluorescence microscopes.

## 1 Introduction

The development of fluorescence microscopy in the 20^th^ century was a major advancement in cell biology, allowing researchers to dynamically observe the intracellular behaviour of specifically labelled molecules and organelles in living cells. However, the spatial resolution of fluorescence microscopy is generally limited by diffraction to ∼200–300 nm; approximately half the wavelength of the illuminating light. As a result, the study of cellular components on a smaller scale was reliant upon electron microscopy. Electron microscopy achieves resolutions down to single nanometres, but is limited by stringent sample preparation requirements to imaging fixed, dead samples.

In the early 21^st^ century, a new family of microscopy techniques emerged: super-resolution microscopy. These techniques ‘break’ the diffraction limit and as such allow for fluorescence imaging at resolutions up to ten times higher than in conventional techniques. The major super-resolution microscopy techniques are: Stimulated Emission Depletion (STED) microscopy (Klar & Hell, 1999), Structured Illumination Microscopy (SIM) (Gustafsson, 2000), and Single Molecule Localisation Microscopy (SMLM) (Patterson et al., 2010). STED microscopy is a confocal laser scanning technique whereby a second, donut-shaped beam is introduced into the illumination path to suppress fluorescence from the periphery of the excitation beam point spread function (PSF). SIM is a widefield technique, which generates interference patterns between the labelled structure in the sample and the periodically patterned (rather than homogeneous) illumination field. From these patterns, an image of the structure can be calculated at a resolution beyond the diffraction limit. SMLM methods include PALM (photo-activated localisation microscopy) (Betzig et al., 2006) and STORM (stochastic optical reconstruction microscopy) (Rust et al., 2006), and involve the large field-of-view acquisition of many thousands of frames of photoswitchable fluorophores under conditions such that only a small, random fraction of the fluorophores are emitting in each frame. Assuming that the emitting fluorophores in each frame are separated by distances greater than the diffraction limit, computational analysis can be used to pinpoint the centres of the emitting molecules with high accuracy and as such a super-resolution ‘map’ of the imaged structure can be generated.

While the above-mentioned super-resolution techniques have established themselves as an important part of cell biology research, the major advantage of super-resolution microscopy, i.e. live-cell compatible imaging, is still not widely exploited. This is due to some fundamental limitations of the techniques. For example, the resolution increase in STED is directly proportional to the intensity of the donut beam (Harke et al., 2008); for resolutions <100 nm this requires the beam to have phototoxic intensity on the order of 0.1–1 GW/cm^2^.Similarly, in SMLM techniques, achieving appropriate photoswitching dynamics of the fluorophores relies on laser intensities on the order of kW/cm^2^, toxic buffers, and UV illumination (Wäldchen et al., 2015). Furthermore, SMLM requires the fluorophores to be stationary over the duration of the acquisition (on the order of minutes and upwards), precluding the imaging of fast dynamic processes and generally complicating any live-cell imaging. Reversible saturable optical linear fluorescence transitions (RESOLFT) microscopy is technique combining the optics of STED microscopy with the fluorophore photophysics of SMLM; here the donut beam facilitates transitions to a long-lived dark state rather than inducing stimulated emission (Hofmann et al., 2005). Although RESOLFT needs several orders of magnitude lower laser intensity compared to STED, there are only a few fluorescent proteins with favourable photoswitching properties for the technique (Wang et al., 2016).Currently, SIM is the only super-resolution technique routinely used for live-cell imaging. However, the resolution of SIM is physically limited to a two-fold resolution increase at best (∼150 nm, in comparison to ∼50 nm and ∼20 nm achievable with STED and SMLM respectively). Methods for achieving higher resolutions in SIM exist, but depend on non-linear excitation of fluorophores only achievable with high, phototoxic illumination intensities (Gustafsson, 2005), or highly specialised hardware inaccessible to the majority of researchers (Li et al., 2015). Of the three main classes of super-resolution microscopy, SMLM offers the highest resolutions with the simplest optical set-up. Therefore, methods for adapting SMLM techniques for live-cell imaging are a particularly active area of research.

## 2 Super-resolution radial fluctuations

One seemingly straightforward solution to increasing the live-cell compatibility of SMLM would be to decrease the intensity of the illuminating laser. However, the photoswitching kinetics of the fluorophores are intimately linked to this laser intensity (Pennacchietti et al., 2017; Dempsey et al., 2011). At high laser intensities the majority of fluorophores are in a dark, non-emissive state, leading to a very small population of molecules emitting in a single camera frame. The majority of SMLM reconstruction algorithms are formulated to work with this high-intensity data, as they rely on mathematical detection of isolated emitted single molecules. The central coordinates of the successfully detected molecules can then be accurately localised (Sage et al., 2015). Lower laser intensities lead to a regime where the majority of the fluorophores are in an emissive state, and so each camera frame contains a larger population of emitting molecules. This larger population increases the likelihood that emitting molecules overlap with each other; such overlapping molecules fail the ‘detection’ phase of analysis and as such are lost from the final image.

**Fig. 1:**
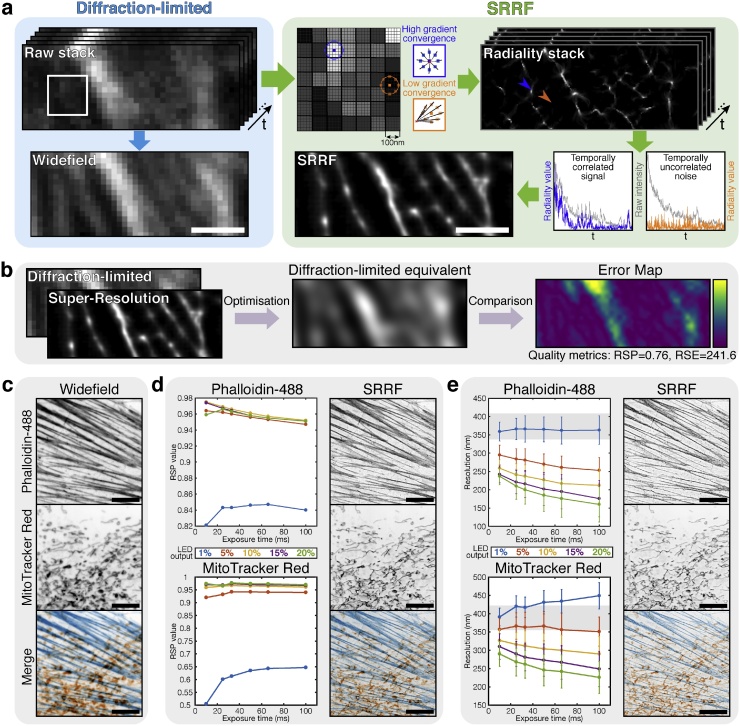
**OVERVIEW OF THE SRRF ALGORITHM AND OPTIMISING SRRF IMAGING.** **a)** Raw data and processing steps in SRRF. The pale cyan box contains the raw, diffraction-limited data series that can be summed to create a single diffraction-limited image. The pale green box contains the SRRF image processing steps. The raw data is split into subpixels (here each pixel from the raw data is split into a 5 × 5 array of subpixels). Examples of the intensity gradients used for the radiality transform are shown for the two highlighted blue and orange subpixels. The blue and orange arrowheads in the radiality stack indicate the location of these subpixels, with their temporal correlations plotted below; coloured plots indicate the radiality variation over time and grey plots indicate the fluorescence intensity variation over time at these locations in the raw data. Scale bar = 1 μm. **b)** Overview of the SQUIRREL algorithm used for optimising SRRF acquisitions. **c)** Widefield LED images of Alexa Fluor 488-phalloidin and MitoTracker Red CMXRos (FluoCells Prepared Slide #1, Invitrogen). **d)** SQUIRREL-calculated RSP (resolution-scaled Pearson’s correlation) values plotted for actin and mitochondria images for different combinations of frame number and exposure time at 5 different LED intensities (indicated as % of maximum output). The highest-quality images (actin: 100 x 10 ms frames at 10% 490 nm LED; mitochondria: 30 x 33 ms frames at 20% 550 nm LED) are displayed alongside. **e)** SQUIRREL-calculated FRC (Fourier Ring Correlation) mean resolution values (error bars = standard deviation of resolution across the entire image) for the same imaging conditions as in **d**. Grey shaded regions indicate the mean ± std. resolutions in the widefield images in **c**. The highest-resolution images (actin: 10 x 100 ms frames at 20% 490 nm LED; mitochondria: 10 x 100 ms at 20% 550 nm LED) are displayed alongside. Scale bars in **c-e** = 10 μm.

Recently, we established a novel analytical approach called Super-Resolution Radial Fluctuations (SRRF) (Gustafsson et al., 2016) that has no detection step and is therefore robust to images containing overlapping fluorophores. The main steps in SRRF image formation are shown in Fig. 1**a**. As in SMLM imaging, the input data is a sequential series of frames of an imaged structure; however, for SRRF analysis there is no requirement for the emitting fluorophores to be sparsely distributed. In most SMLM algorithms, pixels are inspected and a binary decision is made: is this a fluorophore or not? In contrast, SRRF magnifies each pixel into ‘subpixels’, and each subpixel is assigned a non-binary value related to the probability that it contains a fluorophore. To calculate this value, SRRF measures local radial symmetries in the image (dubbed the ‘radiality’ by the authors), which arise from the intrinsic radial symmetry of the microscope PSF. The radiality is a measurement of intensity gradient convergence: for every subpixel, intensity gradient vectors are measured for a ring of nearby surrounding subpixels. The degree of convergence of these vectors at the original central subpixel is then calculated. For a subpixel located close to the true centre of an individual fluorescent molecule, there will be a high degree of convergence and hence a high radiality value (Fig. 1a**, blue box**). For a subpixel displaced from a fluorescent molecule, the surrounding intensity gradients will display low convergence, as they will either be oriented towards the direction of the true molecule location or, in the case of noise, randomly (Fig. 1a**, orange box**). Thus, radiality acts as a proxy for the positions of molecules. By performing this radiality measurement for every subpixel across the input stack of images, a radiality stack is generated.

The radiality transform only considers the spatial information within the images, and so the temporal information within the original data is preserved following transformation. As a result, fluctuations in radiality over time can be analysed to extract further information on the underlying positions of the fluorophores. For example, plotting the radiality value over time shows markedly different temporal fluctuations depending on whether the subpixel is centred on a fluorophore (Fig. 1a**, blue plot**) or not (Fig. 1a, **orange plot**). The degree of temporal correlations across the radiality stack can then be rendered as the final SRRF image. This temporal correlation analysis is similar to that underpinning Super-Resolution Optical Fluctuation Imaging (SOFI) (Dertinger et al., 2009). However, SOFI relies solely on fluorophores possessing distinct fluorescent and non-fluorescent states and does not incorporate additional spatial information. This renders it incapable of increasing the resolution for samples labelled with stable fluorophores such as GFP.

The resolution of the final image will scale with the degree of fluctuations exhibited in the raw data. The raw data displayed in Fig. 1**a** only displays weak temporal fluctuations in comparison to, for example, an SMLM acquisition with distinct blinking fluorophores. Even so, the final SRRF image achieves ∼70 nm resolution (a 5-fold improvement over the widefield resolution of ∼340 nm) as measured by Fourier Ring Correlation (Nieuwenhuizen et al., 2013).

## 3 Optimising SRRF acquisitions

In super-resolution microscopy it is important to be able to evaluate the quality of the generated images. Using our recently-published algorithm SQUIRREL (super-resolution image rating and reporting of error locations) (Culley et al., 2018) we assessed the quality of SRRF images acquired under a range of different imaging conditions. Fig. 1**b** shows the workflow of SQUIRREL and its application to the data displayed in Fig. 1**a**. By inputting a diffraction-limited image and a super-resolution image of the same focal volume, a diffraction-limited equivalent of the super-resolution rendering can be generated. This can then be compared with the true diffraction-limited image and used to highlight ‘error’ regions where the images disagree. For example, the error map in Fig. 1**b** shows that there are mismatches associated with the central bright structure. SQUIRREL also generates quality metrics to allow for comparison of differences between e.g. experimental conditions, acquisition parameters and reconstruction strategies.

Imaging parameters that can affect the quality of a SRRF image include illumination intensity, camera exposure time and the number of frames acquired. Therefore, we used SQUIRREL to optimise SRRF acquisition of a fixed sample labelled with conventional fluorophores and imaged with an LED illumination source. The widefield images of the sample (Alexa 488-labelled actin and MitoTracker Red-labelled mitochondria) are shown in Fig. 1**c**. A series of 1 s raw image sequences with varying exposure times and numbers of frames were acquired for subsequent SRRF reconstruction. Image sequences were acquired with either 100 x 10 ms frames, 40 x 25 ms frames, 30 x 33 ms frames, 20 x 50 ms frames, 15 x 66 ms frames or 10 x 100 ms frames, and these acquisitions were repeated at 5 different LED intensities. All these modalities therefore allow a SRRF frame rate of 1 Hz. SRRF images were reconstructed for each dataset and their quality (Fig. 1d) and resolution (Fig. 1e) measured using SQUIRREL, with the widefield images in Fig. 1**c** used as the references. Both image quality and resolution varied depending on the combination of number of frames, exposure time and illumination intensity, and also varied between the two different fluorophores. Interestingly, for the actin images in particular, the settings yielding the highest resolutions were markedly different from the ones yielding higher qualities.

## 4 Application of SRRF analysis in different microscopes

**Fig. 2:**
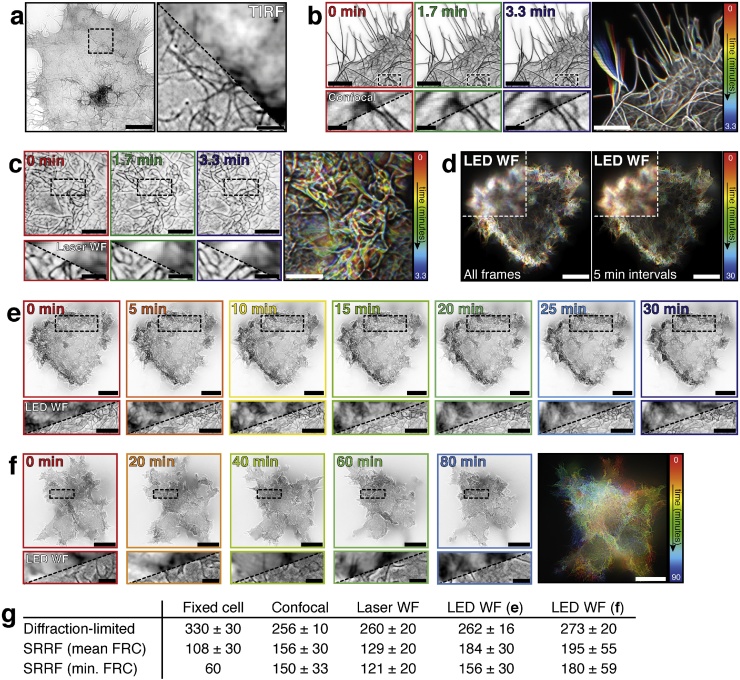
**SRRF IMAGING USING DIFFERENT MICROSCOPES.** **a)** Left: SRRF image of Alexa Fluor-647-labelled phalloidin in fixed Cos7 cells imaged using TIRF with intense laser illumination. Scale bar = 10 μm. Right: enlarged view of the boxed region with the non-super-resolved TIRF image shown. Scale bar = 2 μm. **b)** Greyscale images: individual SRRF time-points (each frame represents 1 s of imaging) of Cos7 cells expressing Utrophin-GFP imaged using confocal microscopy (scale bars = 5 μm). Enlarged views of the boxed region are displayed below as a split between the diffraction-limited confocal image and the SRRF image (scale bars = 1 μm). Coloured image: temporally colour-coded projection of 200 SRRF reconstructions at 1 Hz from 200 s of continuous imaging (scale bar = 5 μm). **c)** Greyscale images: individual SRRF time-points (1 s imaging per reconstructed SRRF frame) of Utrophin-GFP images using widefield laser-based microscopy (scale bars = 5 μm) and enlarged insets showing split between corresponding diffraction-limited images below (scale bars = 2 μm). Coloured image: temporally colour-coded projection on 200 SRRF reconstructions at 1 Hz from 200 s of continuous imaging (scale bar = 5 μm). **d)** Temporally colour-coded projections of SRRF reconstructions of 30 min. of continuous LED-illuminated widefield Utrophin-GFP imaging. Left: projection of all 590 SRRF reconstructions at 0.33 Hz. Right: same dataset, selected SRRF frames at 5 min intervals. Scale bars = 10 μm. **e)** Individual SRRF frames (3 s imaging per time-point) from the projected dataset in the right-hand panel of d) (scale bars = 10 μm), with insets below showing enlarged boxed region split with diffraction-limited LED widefield (scale bars = 5 μm). **f)** Long-term widefield LED timelapse imaging of Utrophin-GFP with SRRF images acquired every 10 min. Greyscale images: individual SRRF frames (3 s imaging per time-point, scale bars = 10 μm) with enlarged insets below showing the diffraction-limited equivalent (scale bars = 2 μm). Coloured image: temporally colour-coded projection of 10 SRRF reconstructions from imaging once every 10 min (scale bar = 10 μm). **g)** Resolutions as measured using the ‘FRC Map’ tool in NanoJ-SQUIRREL. For the fixed cell data, SRRF (mean FRC) is the average resolution across the whole image, with SRRF (min. FRC) representing the best local resolution in the image. For the live cell data, mean FRC is the resolution averaged across all images in a time series, and min. FRC is the average value for the best individual frame within the series. All values are in nm, errors ± SD.

To showcase the versatility of SRRF analysis, we imaged live and fixed samples on a range of microscopes. For a traditional SMLM dataset, we performed *d*STORM imaging (Heilemann et al., 2008) of Alexa Fluor 647-labelled phalloidin-bound actin in a fixed cell and acquired 30,000 frames with high-intensity TIRF illumination. The SRRF reconstruction of this data is shown in Fig. 2**a**. We then performed live-cell imaging of Utrophin-GFP-expressing Cos7 cells using confocal microscopy (Fig. 2b) and widefield microscopy using laser (Fig. 2c) and LED (Fig. 2**d–f**) illumination. In each case, ideal SRRF reconstruction parameters were determined using SQUIRREL to maximise the quality of the final images.

SRRF analysis of confocal microscopy images produces a smaller improvement in resolution compared to widefield imaging modalities (150 ± 30 nm for SRRF compared to ∼200 nm diffraction-limited, measured from the first time-point). For the data shown here, SRRF resolution was most likely limited due to disruptions in the intensity gradients caused by small phase mismatches in the bidirectional laser scanning and non-contiguous time information within each pixel. However, there are few avenues for super-resolution microscopy in confocal microscopes other than STED, which faces challenges when studying live samples as discussed above. SRRF is thus one of the few approaches for breaking the diffraction limit in live-cell confocal microscopy. For example, confocal-SRRF has been used to investigate PIN protein distribution within the plasma membrane of root epidermis cells of *Arabodopsis,* which was unsuccessful with STED due to the rapid bleaching of the fluorescent protein signal (Retzer et al., 2017).

It has been previously demonstrated that SRRF is an ideal approach for increasing the resolution in live-cell laser-based widefield microscopy with conventional fluorophores (Gustafsson et al., 2016; Sirianni et al., 2016). One particularly interesting example of this is in live-cell total internal reflection fluorescence (TIRF) imaging of tubulin tagged with mEos 3.2 via a CRISPR-Cas9 labelling strategy (Khan et al., 2017). The combination of TIRF and SRRF is shown here for live-cell imaging of Utrophin-GFP in Fig. 2c, where the temporally colour-coded projection conveys the dynamics of utrophin filaments captured by SRRF imaging at 1 SRRF frame per second (i.e. 1 Hz). Laser-based SRRF microscopy of conventional fluorophores has also been demonstrated in fixed bacteria for multi-colour imaging in *B. subtilis* (Vega-Cabrera et al., 2017) and z-stack imaging of membrane proteins in *S. aureus* (Weihs et al., 2018).

For the first time here we showcase the application of SRRF analysis to data acquired using live-cell LED-illuminated widefield microscopy. Fig. 2**d** shows temporal colour-coded projections of 30 min of continuous LED imaging, with individual time-points separated in Fig. 2**e**. To date, LED-based live-cell super-resolution microscopy has only been demonstrated once elsewhere, but in a context requiring specialised fluorophores for Förster Resonance Energy Transfer (FRET) (Cho et al., 2013). The compatibility of SRRF analysis with low cost, low intensity LED illumination with GFP-labelled samples allows for simple conversion of routine live-cell imaging experiments into super-resolution data. For example, Fig. 2**f** shows a typical long-term timelapse experiment where a 0.33 Hz SRRF image is acquired once every 10 min. This requires no modification of the microscope or sample; only the acquisition of enough raw frames at each time-point to run SRRF analysis on (here 100 frames at 30 ms exposure). Resolutions for the fixed cell and live-cell data sets are shown in the table in Fig. 2**g**.

## 5 Summary and outlook

We have demonstrated here, in addition to examples present in the literature, that SRRF is a versatile and straightforward method for live-cell super-resolution imaging. It enables routine cell biology imaging experiments to take advantage of super-resolution imaging, without the need for specialised super-resolution microscopes or fluorophores. In the future SRRF will evolve to become more compatible with modern detectors such as sCMOS cameras (Almada et al., 2015) and to benefit from machine learning (Weigert et al., 2018) to enhance its imaging capacity. Furthermore, as SRRF is agnostic to the microscope used for data acquisition, it should be straightforward to combine SRRF analysis with light-sheet microscopy to enable volumetric SRRF imaging. Super-resolution live-cell imaging is set to revolutionise cell biology research, and SRRF will be a considerable part of this effort.

## 6 Materials and methods

### 6.1 Cell lines

Cos7 cells were cultured in phenol red-free Dulbecco’s modified Eagle’s medium (DMEM; Thermo Fisher Scientific) supplemented with 10% (v/v) fetal bovine serum (FBS; Gibco), 1% (v/v) penicillin/streptomycin (Thermo Fisher Scientific) and 2 mM L-alanyl-L-glutamine (GlutaMAX™, Thermo Fisher Scientific) at 37 °C in a 5% CO_2_ incubator.

### 6.2 Sample preparation

For live-cell imaging, Cos7 cells were seeded on ultraclean (Pereira et al., 2015) 25 mm diameter thickness #1.5 coverslips (Marienfeld) at a density of 0.3 – 0.9 × 10^5^ cells/cm^2^. One day after splitting, cells were transfected with a plasmid encoding the calponin homology domain of utrophin fused to GFP (GFP-UtrCH, here Utrophin-GFP) (Burkel et al., 2007) using Lipofectamin 2000 (Thermo Fisher Scientific) according to the manufacturer’s recommendations. Cells were imaged 1–4 days post transfection in culture medium. For phalloidin dSTORM imaging, Cos7 cells were fixed at 37 °C for 15 min with 4% paraformaldehyde in cytoskeleton-preserving buffer (PEM) (80 mM PIPES pH 6.8, 5 mM EGTA, 2 mM MgCl_2_). After fixation cells were permeabilised (1X PEM with 0.25% Triton-X) for 20 min and stained with Phalloidin-AF647 (Molecular Probes, 4 units/mL) for 30 min.

### 6.3 Imaging

*d*STORM Total Internal Reflection Fluorescence (TIRF) imaging of Alexa Fluor 647-phalloidin in fixed cells (Fig. 2a) was performed on a N-STORM microscope (Nikon) using a 100x TIRF objective (Apo TIRF 100x/1.49 Oil, Nikon) and additional 1.5x magnification. Fluorescence emission was collected onto an EMCCD camera (iXon Ultra 897, Andor), yielding a pixel size of 107 nm. 30,000 frames were acquired with 33 ms exposure and 642 nm laser illumination at maximum output power. Drift was estimated using the inbuilt function in NanoJ-SRRF and correction applied during SRRF analysis. *d*STORM imaging was performed in GLOX buffer (150 mM Tris, pH 8, 1% glycerol, 1% glucose, 10 mM NaCl, 1% β-mercaptoethanol, 0.5 mg/ml glucose oxidase, 40 μg/ml catalase) with phalloidin-Alexa Fluor 647 (1 unit/mL).

Epifluorescence laser imaging of Utrophin-GFP in live Cos7 cells (Fig. 2c) was performed at 37 °C and 5% CO_2_ on an Elyra PS.1 inverted microscope (Zeiss) in epifluorescence mode. Images were obtained using a 63x objective (Plan-APOCHROMAT 63x/1.4 Oil, Zeiss) with additional 1.6x magnification which was used to collect fluorescence to an EMCCD camera (iXon 897, Andor), resulting in an image pixel size of 158.7 nm. 20,000 frames were acquired continuously with 10 ms exposure and 488 nm laser illumination at 0.5% of maximum intensity. 100 raw images were used to generate each SRRF image, yielding an effective temporal resolution of 1 s.

Laser-based confocal imaging of Utrophin-GFP in live Cos7 cells (Fig. 2b) was performed on a TCS SP8 scanning confocal microscope (Leica) with a 63x objective (HC PL APO CS2 63x/1.4 Oil, Leica). Fluorescence detection was performed using a Leica HyD hybrid detector in BrightR mode with 10% gain for fluorescence detection using cropping and zoom adjusted to ensure 100 nm image pixels. 488 nm illumination was used at 0.5% of maximum intensity. The pinhole was set to 1 Airy unit and the acquisition time per frame was 20 ms, with 10,000 frames continuously acquired. 50 raw images were used to generate each SRRF image, yielding an effective temporal resolution of 1 s in the SRRF images.

LED-illumination widefield imaging of Utrophin-GFP in live Cos7 cells (Fig. 2**d–f**) was performed at 37 °C and 5% CO_2_ on a N-STORM microscope (Nikon). A 100x TIRF objective (Plan-APOCHROMAT 100x/1.49 Oil, Nikon) with additional 1.5x magnification was used to collect fluorescence onto an EMCCD camera (iXon Ultra 897, Andor), yielding a pixel size of 107 nm. For continuous imaging, frames were acquired for 30 min with 30 ms exposure time and 490 nm LED illumination at 5% of maximum output. 100 raw images were used to generate each SRRF image, yielding an effective temporal resolution of 3 s. For timelapse imaging, settings were as for continuous imaging, except 100 raw frames (30 ms exposure) were acquired once every 10 min with the illumination shutter closed between acquisitions.
